# Editorial for Innovative Artificial Intelligence System in the Children’s Hospital in Japan

**DOI:** 10.31662/jmaj.2025-0076

**Published:** 2025-03-21

**Authors:** Ryuji Hamamoto

**Affiliations:** 1 Division of Medical AI Research and Development, National Cancer Center Research Institute, Tokyo, Japan; 2 Cancer Translational Research Team, RIKEN Center for Advanced Intelligence Project, Tokyo, Japan

**Keywords:** AI hospital, medical AI, deep learning, humanistic medicine

In recent years, expectations for artificial intelligence (AI) technology have increased owing to advances in machine learning technology focused on deep learning. Ever since the introduction of transformer models in 2017 ^(1)^, generative AI technology―represented by products such as ChatGPT―has rapidly evolved, and the use of AI technology is expanding across various fields at a pace that is transforming the social structure of humans. The field of medical AI is no exception―AI-equipped medical device programs have been approved and are being used in clinical practices worldwide, including Japan ^(2)^. The application of AI in medicine has been progressing in three major areas ^(3)^. The first is the analysis of medical images (e.g., endoscopic, radiological, and pathological images) using AI. Most of the research has been conducted in this area, and many results are being applied in clinical practice. Second is the development of tools that use machine learning to analyze multiple medical records, including genetic information, prognosis prediction, lymph node metastasis, and chemotherapy. Third is research using natural language processing, which is a field of AI involved in extracting useful information from electronic medical records and medical studies. The extracted information is applied to medical care, to automatically generate summaries and other documents for reducing the burden of clinical work. This is a field that has grown rapidly with the advent of transformers and is attracting enormous attention in the medical context. Furthermore, research in multi-modal analysis of data from various modalities using AI (such as the integration of natural language processing and medical image analysis) has recently become popular ^(4)^. Because the information available in single modality is limited, multi-modal AI analysis is likely to become prominent in the medical field in the future.

Umezawa et al. ^(5)^ developed an innovative AI system centered on the National Center for Child Health and Development as part of the Japanese Cross-Ministerial Strategic Innovation Promotion Program “Innovative AI Hospital System”. The content includes the use of deep learning technology to accelerate pathological diagnosis using image data, identification of bacterial species, early detection of eye diseases, and prediction of hereditary disorders based on physical characteristics ^(5)^. Moreover, information and communication technology was introduced to diagnose cancer in children, to predict immune responses on the basis of genomic data, and to aid in autism diagnosis by quantifying behavior and communication ^(5)^. The Japanese AI Hospital Project has been promoted with the aim of providing compassionate care to patients while improving the happiness of medical professionals. The application of AI technology in this project to address challenges in pediatric medicine is deemed highly significant.

Importantly, multiple issues must be resolved to promote the use of medical AI in Japan. Among these, I will focus on two points in this discussion. First, AI is not always accurate―even with the recent popularity of generative AI, the issue of hallucinations remains a challenge ^(2)^. Therefore, the introduction of AI into medicine should be performed with extreme caution. I assert that it is important to be cautious about clinical application, and regulatory approval should be obtained after properly evaluating the clinical performance, in addition to developing legal systems and guidelines for medical AI. Second, the importance of creating an environment for sustainable use of medical AI cannot be neglected. Although it is beneficial that large budgets are being allocated for national projects, it is necessary to standardize the path that each medical institution can maintain sustainably thereafter. Utilization of AI in medical insurance is a critical issue, but developments regarding this in Japan are lacking. Moreover, given the current situation of the medical insurance system in Japan, there are some difficulties in relying solely on insurance-covered medical care. Therefore, I believe that it is necessary for the industry, government, and academia to work together in creating an environment that can completely reveal the benefits of medical AI within the framework of a larger medical system.

Considering the potential capabilities of AI, the clinical applications of medical AI will undoubtedly progress in the future. Furthermore, continuous efforts are required to resolve the aforementioned issues; it is also important to promote the sound applications of AI in medicine, with the aim of maximizing the benefits to patients while placing importance on the idea of “humanistic medicine.”

## Article Information

### Conflicts of Interest

None
